# Controversy: Synthetic Hairs and their Role in Hair Restoration?

**DOI:** 10.4103/0974-7753.66913

**Published:** 2010

**Authors:** Venkataram Mysore

**Affiliations:** Center for Advanced Dermatology, Bangalore, India

**Keywords:** Artificial hair fibers, alopecia, FDA, hair restoration, synthetic hairs

## Abstract

The subject of artifical hair fibers is controversial, in view of their chequered history and the ban by federal drug administration (FDA) on their use. This article analyzes different aspects of their use.

## INTRODUCTION

The use of artificial hair fibers in hair restoration is a controversial one. While the product companies market it as a simple, cosmetically effective remedy, the product has not found wide acceptance by physicians because of its chequered history and lack of proper evidence. The fact that it is a banned item in many countries has further put legal questions on its use. Physicians are asked by prospective patients about its use and therefore awareness about the product is important. This article outlines the pros and cons of using such a product.

## ARTIFICIAL HAIR FIBER – HISTORY

In 1983, federal drug administration (FDA) in USA banned the fibers and the reasons for the ban were cited as follows:

1. The fibers presented RISKS of illness or injury due to nonbiocompatibility of the fibers and nonmedical performance of the implant.
2. The fibers presented FRAUD due to:
  - spreading of deceptive information on the efficacy of result
  - inadequate information on risks deriving from implant
  - they did not show any benefit for public health

(The Ban on Prosthetic Hair Fibers established in Section 895.101 of 21 Code of federal regulations of FDA title 21 vol. 8 revised as of 01 April, 2004)

The reasons which prompted such a ban were the numerous side effects which were reported.[1–6] These included:

1. recurrent infections
2. rejection and periodic loss of fibers needing frequent replacement
3. frequent allergic reactions leading to severe contact dermatitis, irritant effects
4. fears about possible carcinogenicity
5. cicatricial alopecia
6. granulomatous hypersensitivity
7. cyst formation

However, the companies continued to market the product in other countries and presently two products are available:

1. Biofibre from Medicap Italy available since 1996 and
2. Nido corporation from Japan since 1999.

Biofibre has received CE certification in Europe and has reapplied for permission by FDA and the application is pending.

The companies claim that many of the previous problems associated with the fibers have been sorted out and hence the fibers can be used safely. Previously, synthetic fibers made of materials such as monacrylic, polyacrylic and polyester were marketed as were natural fibers, such as processed human hair. Currently available synthetic hair fibers consist of polyamide material (Biofibre CE0373), which has been claimed to be inert and safe. They are implanted in an easy and a simple technique into the galea by a knot through an implanter device. The advantages claimed are the relative ease of the procedure which can be learnt in few days, the technique being relatively bloodless and is with immediate cosmetic result. However, in contrast to transplants, these fibers do not grow and hence cannot be cut or shaven. A study in 1995[6] stated “despite an apparently improved complication rate, the new technique of hair fiber implantation remains a doubtful procedure and cannot be recommended in view of possible permanent sequelae”.

## IS THERE EVIDENCE TO SUPPORT THEIR USE?

A Medline search (accessed on 01 February, 2010 using key words artificial hairs; synthetic hair; prosthetic hair) showed only four reports supporting these claims.[7–10] One report [8] assessed a large number of patients (196) with a 2-year follow-up and found that clinical subjective and photographic objective judgment, evaluation of Hamilton scale grading and covered area rate, show very satisfactory improvements. Relevant adverse events were limited to 1.02%. The study concluded that careful medical follow-up with regular scalp check-up minimizes complications to a very acceptable rate and overall results are definitely satisfying. One report has documented their use in scars.[9] Another recent publication[10] found the yearly fall rate to be 15–20%, but included a very small number (10) of patients.

Thus, the level of evidence available is poor. Further before these studies also documented some side effects such as recurrent mild folliculitis (in 30% of patients) and an annual fiber fall rate of 15–20%.

## OPINION OF ASSOCIATIONS AND SOCIETIES

The matter was considered by the International society of hair restoration. After considering all aspects of the subject, the society stated that “The International Society of Hair Restoration Surgery does not voice an official position with regard to the use of artificial hair fibers and leaves their use up to the regulatory authority within that country. It is the view of the Society that this is a surgical procedure and as such should be confined to active participation of an experienced, licensed medical doctor in a reputable medical clinic or university setting. As with any surgical procedure, complications may occur which should be handled under a physician’s care” (Adopted by the Board of Governors, 08 November, 2004 at www.ishrs.org.

A recent publication on guidelines in esthetic practice in Singapore, listed all aesthetic practices as cat A (with high evidence) and list B (poor evidence). Significantly, artificial hair was not included even in list B.[^11^]

Thus, it is obvious that there are serious limitations and constraints with respect to the use of the topic. It is also important to note that safe and effective options are now available for hair restoration. Both follicular unit transplantation and follicular unit extraction have revolutionalized the field and it is possible for trained hands to give very satisfactory results in most cases. However, being surgical procedures, they demand proper training and experience. These surgeries also need a proper team to perform the procedures. These factors have led some physicians to choose the option of artificial hairs. However, such a choice in view of the evidence presented above, raises several questions, both ethical and legal:

1. Should physicians choose an option only because it is easy?
2. Is it not the job of doctors to choose the correct option with proper evidence?
3. Is it not their job to learn techniques even if they are difficult?
4. Is it ethically correct to use a banned item when surgical hair restoration is available?

## IS THERE ANY INDICATION AT ALL FOR USING THESE FIBERS?

These arguments do not of course propose that we should close our options with respect to the use of such fibers. Definitely more research, through further controlled studies, needs to be performed as they can represent an option in certain selected situations, such as patients without adequate donor hair, patients with cicatricial alopecia, scars, possibly Alopecia Areata, etc.

However, such use should be considered as an experimental approach and therefore should receive approval by ethical committees and adhere to all guidelines and standards needed for a scientific trial. Also, such studies should be conducted only after prior informed consent from the patients about all aspects of the treatment to patients. More importantly, the study should be conducted without charging any fee to patients, as is done in any drug trial.
